# Pharmacokinetic Study of a Novel Antihyperlipidemic Agent LM-13765- A Prodrug

**DOI:** 10.4103/0250-474X.59546

**Published:** 2009

**Authors:** T. M. Shah, S. S. Savle, M. T. Chhabria, I. S. Rathod, C. J. Shishoo, P. S. Brahmkshatriya

**Affiliations:** Department of Pharmaceutical Chemistry and Department of Quality Assurance, L. M. College of Pharmacy, Navrangpura, Ahmedabad-380 009, India

**Keywords:** Antihyperlipidemic agent, LM-13765, pharmacokinetics, HPTLC, prodrug

## Abstract

A sensitive and specific high performance thin layer chromatographic method has been developed for estimation of a novel antihyperlipidemic agent LM 13765 in rabbit plasma and its use for pharmacokinetic study has been evaluated. The proposed method was employed to study pharmacokinetics of LM 13765 in rabbits. It was observed that LM 13765 metabolized immediately after oral administration. The metabolite of LM 13765 was identified and characterized as LM 13765-C. A sensitive and specific HPTLC method was developed for estimation of LM 13765-C in plasma after oral administration of LM 13765 and pharmacokinetic parameters were determined. Biological screening of LM 13765-C on hyperlipidemic rats indicated that it is less potent than the parent compound which is indicative of biotransformation of LM 13765 to active form LM 13765-C.

N-Cyanovmylamidine a useful intermediate for pyridine synthesis have been successfully isolated from our laboratory[1]. Earlier, we have reported synthesis and antihyperlipidemic activity in a series of novel N-cyanovinyl formamidines, where some of the compounds were found to possess potent antihyperlipidemic activity[2]. Among these compounds, N-[2-carbethoxy-2-cyano-1-(*p*-chloroanilino)vinyl]formamidine, LM 13765 (fig. 1) was found to be more potent than the standard drug gemfibrozil in terms of lowering of serum cholesterol and triglycerides and elevating the serum HDL levels. Acute toxicity study of LM 13765 has shown no mortality or behavioral changes in mice after oral administration up to the dose of 4.5 g/kg. Promising results obtained from the pharmacological screening and acute toxicity studies prompted us to study pharmacokinetic properties of LM 13765.

**Fig. 1:**
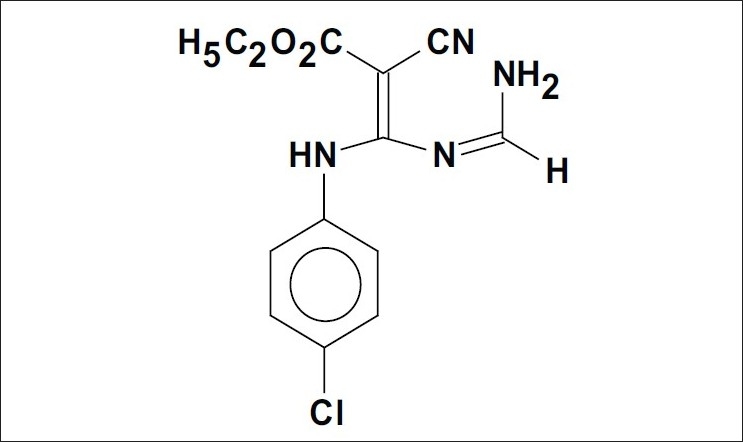
Chemical structure of LM-13765

High performance thin layer chromatography (HPTLC) has been used extensively for analysis of various chemicals, drugs and dosage forms[3–7]. The present study describes development of sensitive and specific HPTLC methods for estimation of levels of LM 13675 and its active metabolite LM 13765-C in rabbit serum, validation of these methods and their application in pharmacokinetic study of LM 13765 and LM 13765-C in rabbits.

## MATERIALS AND METHODS

Compound LM 13765 and LM 13765-C were synthesized, purified and characterized in the laboratory. Acetonitrile, benzene, methanol, sodium carbonate, anhydrous sodium sulphate (S. D. Fine Chemicals, Boisar, India) and concentrated hydrochloric acid (HCl) (35% v/v, Ranbaxy Fine Chem Ltd., SAS Nagar, India) were of analytical reagent grade. TLC aluminum sheets pre-coated with silica gel 60 F_254_ (layer thickness 0.2 mm, 10×10 cm, E. Merck, Dramstadt, Germany) were used as stationary phase.

An HPTLC system (Camag Sonnenmattstr., Muttenz, Switzerland) consisting of Camag Linomat IV semiautomatic spotting device, Camag twin-trough TLC chamber (10×10 cm), Camag TLC scanner 3 and Camag CATS4 software and a 100 μl Hamilton syringe (Hamilton company, NV, USA) were used for analysis.

### Preparation of solutions:

A stock solution of LM 13765 was prepared in acetonitrile at a concentration of 100 g/ml. The stock solution was further diluted with acetonitrile to obtain standard solution of LM 13765 at the concentration levels ranging from 10 to 60 μg/ml. A working standard solution of LM 13765 (40 μg/ml) was prepared by adequate dilution of the stock solution with acetonitrile. A stock solution of LM 13765-C was prepared in methanol at a concentration of 100 μg/ml. The stock solution was further diluted with methanol to obtain working standard solution of LM 13765-C having the concentration of 10 μg/ml.

### HPTLC method for estimation of LM 13765:

Drug/metabolite-free plasma or plasma spiked with fixed aliquots of LM 13765 or rabbit serum sample (0.2 ml) was taken in a glass centrifuge tube (15 ml capacity). Sufficient amount of acetonitrile was added to make the volume to 1.6 ml and vortexed at high speed for 1 min. The mixture was centrifuged (2500 rpm, 15 min) and the clear supernatant layer was transferred to another tube and dried over anhydrous sodium sulphate (0.25 g). Adequate volumes of the dried acetonitrile layer were spotted onto the TLC plate. Chromatographic estimations were performed using precoated silica gel 60 F_254_ TLC plates (prewashed with methanol and dried in air) under following conditions; A mobile phase, benzene:methanol (8:2 v/v); chamber saturation time, 60 min; temperature, 25±2°; development of plates, ascending mode; migration distance, 50 mm; slit dimension, 3×0.3 mm; wavelength of detection, 314 nm; band width, 4 mm; space between two bands, 4 mm; spraying rate, 10 μl/s.

Suitable volumes of standard solution of LM 13765 in acetonitrile or extracts of drug/metabolite-free plasma or rabbit serum samples, were applied on TLC plate under nitrogen stream using semiautomatic spotter. The plate was dried in air and developed at constant temperature (25±2°) using a mixture of benzene:methanol (8:2 v/v) as the mobile phase. After development, the plate was dried under a stream of hot air. Photometric measurements were performed at 314 nm in the reflectance mode with Camag TLC Scanner 3 connected through a computer running Camag CATS 4 software. Peak area values were used for quantitative determination.

Aliquots of 50, 100, 200, 300 and 400 μl of working standard solution (40 μg/ml of LM 13765) were mixed with 0.2 ml of drug/metabolite-free plasma. Sufficient amount of acetonitrile was added into each tube to make the volume up to 1.6 ml and extracted as described under extraction of LM 13765. Ten microlitres of the extracts were spotted onto the TLC plate to obtain the concentration range of 12.5 to 100 ng/spot for LM 13765. Calibration curves were constructed by plotting peak areas of LM 13765 against respective concentrations.

The method was validated in terms of linearity, limit of quantitation, limit of detection, precision, accuracy and specificity. The linear response for LM 13765 spiked in plasma, was determined by analyzing corresponding samples five times for each concentration in the range of 12.5 to 100 ng/spot. The lowest concentration of the calibration range is taken as the limit of quantitation for LM 13765 while the limit of detection was determined by spotting concentrations of LM 13765 lower than the limit of quantitation. The precision of the analysis, in terms of variability in the peak area, was established over the entire calibration range by analyzing plasma samples spiked with LM 13765 daily for 5 d over a period of 1 w. The recovery of LM 13765 from plasma (extraction efficiency) was determined by comparing peak areas obtained from plasma spiked with LM 13765 at concentration levels of 12.5, 25, 50, 75 and 100 ng/spot with the corresponding standards of LM 13765. Accuracy of the proposed method was determined by standard addition method at three different concentrations of LM 13765 in the range of 12.5 to 100 ng/spot. Specificity of the proposed method was assessed in terms of purity of chromatographic peak corresponding to LM 13765 by correlating the spectra scanned at peak start, peak apex and peak end positions of the spot corresponding to LM 13765. Stability of LM 13765 in plasma under storage conditions (-20°), was studied by analyzing spiked plasma sample (0.2 ml) containing 5 μg of LM 13765. The sample was analyzed, on the same day as well as after 24 h storage, for the amounts of LM 13765.

### Preliminary *in vivo* study:

In a preliminary study, three healthy rabbits (New Zealander strain) of either sex weighing 1.4-2.5 kg were selected. They were housed under standard conditions for a week. The experiment was performed as per the guidelines of Institutional Animal Care Committee constituted as per the guidelines of the CPCSEA) and the protocol was duly approved by the Institutional Animal Ethics Committee.

Animals were deprived of food for 12 h before administration of the study compound LM 13765, while water was allowed *ad libitum*. Each rabbit was administered a dose of 100 mg/kg of LM 13765 by oral route in the form of an aqueous suspension containing sodium carboxymethyl cellulose (1% w/v) as a suspending agent. Blood samples (2 ml) were collected from the marginal ear vein before administration of drug and at the intervals of 0.5, 1, 1.5, 2, 4, 6, 8, 10 and 12 h. The samples were allowed to stand for 1 h and then centrifuged at 5000 rpm for 25 min to separate the serum. Serum, thus separated, was collected in clean dry glass tubes and immediately subjected to analysis using the proposed HPTLC method for estimation of LM 13765.

### Pilot pharmacokinetic study:

Five healthy rabbits (New Zealander strain) of either sex weighing 1.4-2.5 kg were selected. They were housed for a week and were used to study the pharmacokinetics of LM 13765, in terms of plasma levels of LM 13765-C, after oral administration of LM 13765 (100 mg/kg body weight) following the similar protocol as described under the preliminary *in vivo* study. The pharmacokinetic parameters were calculated using a model-independent method. The peak level (C_max_) and the time taken to reach peak level (t_max_) were observed. The elimination rate constant (K_el_) and the terminal elimination half-life (t_1/2_) were estimated by linear regression of the terminal part of the log concentration-time curve. The area under the plasma concentration-time curve (AUC) was determined by linear trapezoidal rule and extrapolated to identify AUC_0-α_ by dividing the last measurable concentration by the elimination rate constant.

### HPTLC method for estimation of LM 13765-C:

Extraction of compound LM 13765-C was carried out in a manner similar to that of LM 13765 with following modifications: The sample was acidified with 0.2 N HCl (300 μl), mixed by vortexing at high speed. The sample was heated on water bath (80°, 10 min). After bringing down to room temperature, the pH of the sample was adjusted to 7.4 by addition of sodium carbonate solution (10% w/v, 350 μl). The mixture was extracted with benzene (2×1 ml) instead of acetonitrile. Dried benzene extract was evaporated to dryness at room temperature by flushing with nitrogen gas. The residue was reconstituted in acetonitrile (200 μl) and adequate volumes were spotted onto the TLC plate.

Chromatographic conditions were kept similar to that of LM 13765 with following modifications: Mobile phase composition: toluene:methanol (9.2:0.8 v/v) and wavelength of detection: 274 nm. Chromatographic analysis was carried out in a similar way as LM 13765. Calibration curve was prepared by a method analogous to that used for LM 13765 with following modifications; Aliquots taken: 10, 25, 50 and 75 μl of working standard solution (10 μg/ml of LM 13765-C in methanol). Forty microlitres of the reconstituted samples were spotted onto the TLC plate to obtain the concentration range of 20 to 150 ng/spot for LM 13765-C.

Validation of HPTLC method was done by a method similar to that used for LM 13765 with following modifications: Concentration levels taken for recovery study: 20, 50, 100 and 150 ng/spot.

### Preliminary *in vivo* study after oral administration of LM 137765-C:

In a preliminary study, three healthy New Zealand rabbits of either sex weighing 1.4-2.5 kg were selected. The animals were deprived of food for 12 h before administration of the study compound LM 13765-C, while water was allowed *ad libitum*. Each rabbit was administered a dose of LM 13765-C equivalent to 100 mg/kg of LM 13765 by oral route in the form of an aqueous suspension containing sodium carboxymethyl cellulose (1% w/v) as a suspending agent. Blood samples (2 ml) were collected from the marginal ear vein before administration of drug and at the intervals of 0.5, 1, 1.5, 2, 4, 6, 8, 10 and 12 h. The samples were allowed to stand for 1 h and then centrifuged at 5000 rpm for 25 min to separate the serum. Serum, thus separated, was collected in clean dry glass tubes and immediately subjected to analysis using the proposed HPTLC method for estimation of LM 13765-C. The pharmacokinetic parameters were calculated using a model-independent method. The peak level (C_max_) and the time taken to reach peak level (t_max_) were observed. The elimination rate constant (K_el_) and the terminal elimination half-life (t_1/2_) were estimated by linear regression of the terminal part of the log concentration-time curve. The area under the plasma concentration-time curve (AUC) was determined by the linear trapezoidal rule and extrapolated to identify AUC_0-α_ by dividing the last measurable concentration by the elimination rate constant.

## RESULTS AND DISCUSSION

Because of the good solubility of LM 13765 in acetonitrile, it was selected as a solvent, which could also provide sample clean-up by precipitating the proteins. It was observed that among the various mobile phases tried, a mixture of benzene:methanol (8:2 v/v) resolved the analyte (R_f_= 0.21) from the endogenous plasma components. Pre-washing of the plates with methanol and pre-saturation of the chamber with mobile phase for 60 min resulted in sharpening of the bands and better resolution. The photometric estimations were performed at 314 nm, which represented the wavelength of maximum absorption.

It was observed that the peak area response for LM 13765 spiked in plasma increases linearly with its concentration in the range of 12.5 to 100 ng/spot. Linear regression of the calibration curve data indicated a good correlation with a correlation coefficient of 0.997. The limit of quantitation was found to be 12.5 ng/spot while the limit of detection was found to be 10 ng/spot for LM 13765. Thus the method was found to be sensitive. The precision of the analysis (% CV) was found to vary from 4.31 to 7.89 over the concentration range of the calibration curve (Table 1). The extraction method employing acetonitrile exhibited very good recovery (> 90% of LM 13765 extracted from the matrix) of LM 13765 spiked in plasma. The method was found to be accurate with the accuracy ranging from 93.0 to 102.1% over the range of concentrations studied (Table 2). Comparison of the spectra scanned at peak start, peak apex and peak end positions of the spot corresponding to LM 13765 has shown a very good correlation (r = 0.999) indicating the purity of the spot. Thus, there was no interfering components eluting at the R_f_ corresponding to LM 13765, and hence, indicating that the method is specific for analysis of LM 13765. Various validation parameters for the proposed HPTLC method for estimation of LM 13765 in plasma/serum are summarized in Table 3. However, the stability study of LM 13765 in plasma indicated that under storage conditions (-20°) LM 13765 undergoes significant degradation in one day. Thus, it is necessary that immediately after collection of blood samples and separation of serum, the serum samples should be subjected to extraction followed by analysis. Thus, the proposed method was found to be simple, sensitive, accurate, precise and specific for estimation of plasma/serum levels of LM 13765 and was employed for monitoring the levels of LM 13765 after oral administration in rabbits.

**TABLE 1:** RECOVERY OF COMPOUND LM 13765 FROM PLASMA

Concentration of drug added (ng/spot)	Concentration of drug detected^a^ (ng)	% Recovery	% CV^b^
12.5	11.81±0.93	94.48	7.89
25	22.41±1.09	89.64	4.51
50	48.63±5.25	97.26	10.7
75	66.66±3.03	88.88	4.83
100	88.21±3.8	88.21	4.31

a mean±SEM (standard error of mean), n=4.

b CV= coefficient of variation

**TABLE 2:** LINEARITY

Concentaration of drug added (ng)	Area^a^	% CV^b^	% Accuracy
12.5	1270.9±168.4	13.29	102.1
25	2170±114.6	5.27	100.9
50	3464.7±146.3	4.22	102.1
75	4222±84.1	1.99	98.4
100	5412.8±374.8	6.92	93.0

a mean±SEM (standard error of mean), n=5.

b CV= coefficient of variation

The plasma/serum samples collected in the preliminary *in vivo* study after oral administration of LM 13765 to rabbits, when analysed by the proposed method, no spot corresponding to LM 13765 (R_f_= 0.21) was observed. Instead, all these samples exhibited a new spot eluting the R_f_ of 0.57. This fact indicated that LM 13765 might be getting converted, under physiological conditions after oral administration, to a new compound having R_f_ value of 0.57. Therefore, it was thought of interest to establish the identity and characterize this compound (metabolite). It was, thus, inevitably essential to develop a separate HPTLC method for estimation of serum levels of the metabolite, validate it and utilize this method to study pharmacokinetic behavior of LM 13765 after oral administration.

**Scheme 1:**
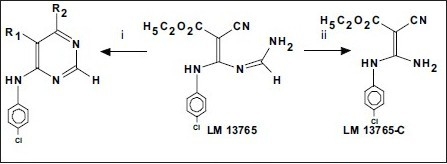
Chemical structure of metabolites of LM-13765 and their route of synthesis LM13765-A; R_1_ = CN; R_2_ = OH; i) NaOEt/EtOH; LM13765-B; R_1_ = CO_2_C_2_H_5_; R_2_ = C_1_; i) Dry HCl; LM 13765-C; ii) HCl/Water-Methanol

The probable structures of metabolites of LM 13765 and the routes of their synthesis have been shown in Scheme 1. The chemical nature of these metabolites can be represented as 1) cyclised pyrimidines: i) 5-cyano-6-(*p-*chloroanilino)pyrimidin-4(3H)-one, LM 13765-A, or ii) 5-carbethoxy-4-chloro-6-(*p-*chloroanilino)pyrimidine, LM 13765-B, and 2) The hydrolytic decomposition product ethyl 3-amino-2-cyano-3-(*p*-chloroanilino)acrylate (aminal), LM 13765-C. Compounds LM 13765-A, LM 13765-B and LM 13765-C were synthesized and characterized by various spectral analysis like UV, IR, ^1^H NMR, mass and elemental analysis.

For identification of the metabolite, its R_f_, UV spectrum and IR spectrum were compared with similar characteristics of LM 13765-A, LM 13765-B and LM 13765-C. Interestingly, it was observed that the metabolite exhibited identical R_f_, UV and IR spectral characteristics to that of LM 13765-C, suggesting that LM 13765 may undergo hydrolytic degradation to its aminal, LM 13765-C in the body immediately after oral administration (within 0.5 h). Thus, the metabolite appearing in serum was identified as LM 13765-C. Further, the lipophilicity (ClogP) of both, the parent compound vinylamidine, LM 13765 and the aminal, LM 13765-C, was determined and the corresponding values were found to be 2.3 and 1.4, respectively. With this background a HPTLC method was developed for determining the plasma levels of LM 13765-C.

**Fig. 2:**
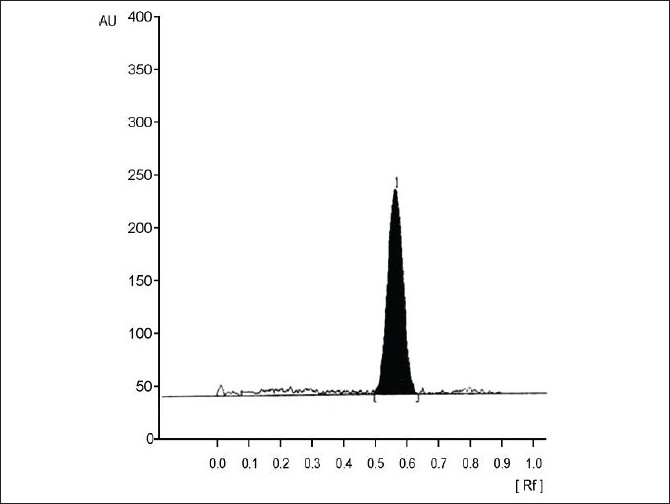
Chromatogram showing separation of LM 13765-C AUC is area under the curve and Rf is the retention factor

**Fig. 3:**
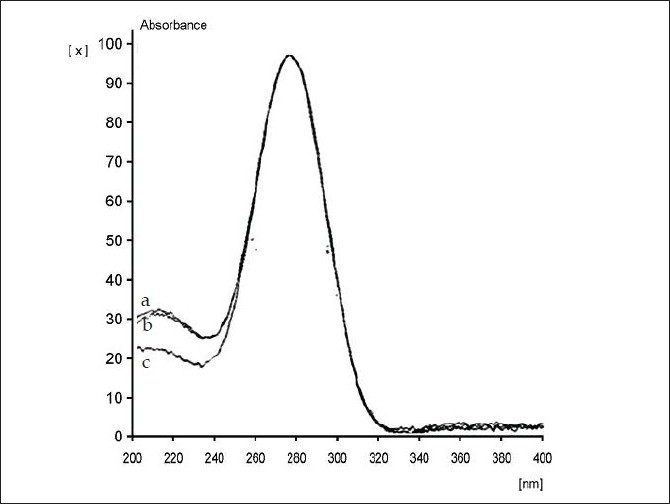
Chromatograms for a) drug/metabolite-free plasma; b) LM 13765-C spiked in plasma and c) standard LM 13765-C Overlay chromatograms of drug/metabolite-free plasma, LM-13765-C spiked in plasma and standard LM-13765-C

Precision of the analysis (% CV) was found to vary between 2.91 to 5.85 over the concentration range studied (Table 3). The extraction method employed exhibited very good recovery (>90% of LM 13765-C extracted from serum sample) of LM 13765-C spiked in plasma (fig. 2). The method was found to be accurate with the accuracy ranging from 93.83 to 108.48% over the concentration range of 20 to 150 ng/spot (Table 3). Comparison of the spectra scanned at peak start, peak apex and a peak end position of the spot corresponding to LM 13765-C has shown a very good correlation (r = 0.999) (fig. 3), indicating the purity of the spot. Thus, there was no interfering component eluting at the R_f_ corresponding to LM 13765-C, further indicating that the method is specific for analysis of LM 13765-C. Various validation parameters for the proposed HPTLC method for estimation of LM 13765-C in plasma/serum are summarized in Table 3. Further, it was observed that LM 13765-C is stable under the storage conditions (-20°) for one day. Thus, the proposed method was found to be simple, sensitive, accurate, precise and specific for estimation of plasma/serum levels of LM 13765-C and was employed for monitoring the levels of LM 13765-C after oral administration of LM 13765 as well as LM 13765-C in rabbits.

**TABLE 3:** SUMMARY OF VALIDATION PARAMETERS FOR THE PROPOSED HPTLC METHOD IN PLASMA

Parameter	LM 13765	LM 13765-C
Linearity range (ng/spot)	12.5-100	20-150
Precision (% CV)	4.31-7.89	2.91-5.85
% Accuracy	92.98-102.06	93.83-108.48
Limit of detection (ng/spot)	12.5	10
Limit of quantification (ng/spot)	10	20
Specificity	Specific	Specific
Average extraction efficiency	97.3 %	67.62%
(n = 5)		

**Fig. 4:**
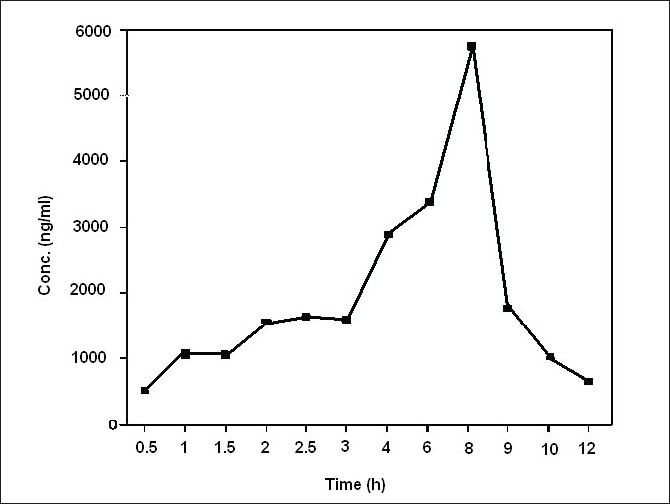
Average serum concentration-time profile of LM 13765-C after oral administration of LM 13765 Average serum concentration-time profiles of LM 13765-C after oral administration of LM 13765 to rabbits (n=5)

**Fig. 5:**
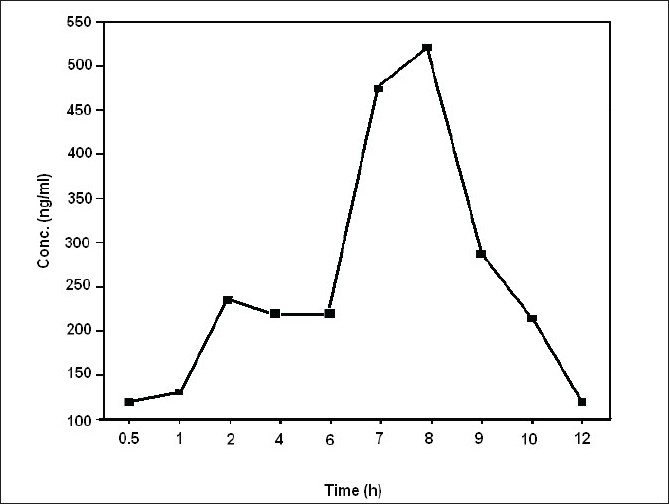
Average serum concentration-time profile of LM 13765-C after oral administration of LM 13765-C Average serum concentration-time profile of LM 13765-C after administration of LM 13765-C to rabbits (n=3)

Average serum concentration-time profile of LM 13765-C after oral administration of LM 13765 is shown in fig. 4. Different pharmacokinetic parameters of LM 13765 are summarized in Table 4. Biological screening of LM 13765-C indicated that it possesses very good antihyperlipidemic activity but is less potent than the parent compound, LM 13765. Therefore, it was of equal importance to understand the pharmacokinetic behavior of LM 13765-C after oral administration. Therefore, *in vivo* study was conducted where the rabbits were administered LM 13765-C by oral route and the serum levels of LM 13765-C were monitored using the proposed HPTLC method for LM 13765-C. Average serum concentration-time profile of LM 13765-C after oral administration of LM 13765-C is shown in fig. 5. Pharmacokinetic parameters of LM 13765-C are also summarized in Table 4. Comparison of various pharmacokinetic parameters obtained after administration of LM 13765 and LM 13765-C indicated that on administration of LM 13765, higher values for C_max_ and AUC_0-12_ are obtained than those after administration of the aminal, LM 13765-C. On the other hand, in both the cases the values for t_max_, K_el_ and t_1/2_ are comparable (Table 4). Lower C_max_ and AUC_0-12_ values after administration of LM 13765-C suggests that it may be poorly absorbed from gastrointestinal tract (GI tract) as compared to the parent compound, LM 13765. This could be the reason for the comparatively less potent antihyperlipidemic activity of LM 13765-C as compared to LM 13765. The comparatively poor absorption (both rate, C_max_, and extent of absorption, AUC_0-12_) of LM 13765-C, as compared to LM 13765, from GI tract can be explained on the basis of their ClogP values also, which indicate that LM 13765 (clogP = 2.3) is more lipophilic than LM 13765-C (clogP = 1.4). Thus, the fact that immediately after administration (within 0.5 h) of LM 13765, its metabolite starts appearing in the serum, indicates that the compound might be undergoing hydrolytic cleavage after oral absorption into an active metabolite, LM 13765-C. Therefore, the results suggest that LM 13765 may be acting as a prodrug with better absorption from GI tract after oral administration which completely gets metabolized into LM 13765-C immediately after oral administration, and exhibits potent antihyperlipidemic activity.

**TABLE 4:** PHARMACOKINETIC PARAMETERS OF COMPOUNDS LM 13765 AND LM 13765-C

Compound	C_max_ (ng/ml)	T_max_ (h)	Auc_0-12_ (ng.h/ml)	K_el_ (1/h)	T_1/2_ (h)
LM 13765^a^	5790.8±832.6	8	25479.4±2854.9	1.52	5.12
LM 13765-C	288.5±23.11	8	1526.8±140.1	1.45	4.81

a C_max_, t_max_ and AUC observed in compound LM 13765 were measured as compound LM 13765-C as compound LM 13765 was found to get metabolized to its aminal LM 13765-C.
